# Usability and acceptability of a power tool with electronic depth gauge for orthopedic drilling – a preclinical randomized controlled trial in sawbones

**DOI:** 10.1007/s00402-025-05839-3

**Published:** 2025-04-11

**Authors:** Jacob Schade Engbjerg, Rikke Thorninger, Christian Lind Nielsen, Juliane Rinne, Jan Duedal Rölfing

**Affiliations:** 1 Department of Orthopaedics, Regional Hospital Randers, Randers, Denmark; 2 MidtSim, Department of Clinical Medicine, Aarhus University, Central Denmark Region, Aarhus, Denmark; 3 Department of Orthopaedics, Aarhus University Hospital, Aarhus, Denmark

**Keywords:** Orthopedic skills, Automatic depth measurement, Screw length determination, Depth gauge, System Usability Scale

## Abstract

**Introduction:**

Orthopedic drilling and screw placement require precision to obtain the effect of bicortical screwing and avoid complications such as soft tissue damage. Traditional manual depth gauges, while effective, are prone to human error. This study evaluates the usability and acceptability of an automated electronic depth gauge compared to a conventional manual depth gauge in a preclinical setting.

**Methods:**

A randomized controlled cross-over trial was conducted with 45 participants of varying clinical experience (12 medical students, 11 resident doctors, and 22 consultants, 14/22 were senior consultants) at Aarhus University Hospital. According to randomization, participants used either the “electronic -> manual” or “manual -> electronic” depth gauge and subsequently the sequence “with -> without” or “without -> with” soft tissue protector to measure screw length in sawbones. Efficiency was assessed by measuring time (savings), and usability was evaluated using the System Usability Scale (SUS).

**Results:**

The electronic depth gauge significantly reduced the time required to measure screw length, with medical students saving the most time, p < 0.001. 65/331 (20%) of screws were changed, with no difference in the rate of screw changes between manual and electronic depth gauges, p = 0.76. Level of experience was significantly correlated with the rate of screw changes, p = 0.03. Resident doctors changed fewer screws than medical students, with no difference between the remaining groups. The median SUS score was 87.5, indicating excellent usability. Most participants rated the tool as “best imaginable” or “excellent.”

**Conclusion:**

The electronic depth gauge was time-efficient and surgeons rated it as highly usable. However, screw exchanges were not significantly minimized in this study, which might be mitigated by proper training and awareness of device-specific recommendations. The effect of electronic depth gauges on screw exchange rates and its clinical applicability warrents further research.

**Supplementary Information:**

The online version contains supplementary material available at 10.1007/s00402-025-05839-3.

## Introduction

Drilling, measuring and deciding on the appropriate screw length are essential skills in orthopedic surgery [1–3]. Surgeons rely on tactile, proprioceptive, and auditory feedback to gauge the depth which can be subjective and vary between individuals [4–6].

The precise placement of screws is a critical aspect of orthopedic surgery and internal fixation [2, 3]. This process requires accurate measurement of bone depth to prevent excessively long screws and complications such as soft tissue irritation/damage or too short screws with suboptimal fixation [1, 3]. Traditional methods for determining screw length involve manual depth gauges, which are prone to human error and variability in measurement [2, 3, 6]. Moreover, manual depth measures can take significant time, especially in osteoporotic bone or comminuted fractures. The introduction of electronic depth gauges promises to enhance accuracy and usability [4], and potentially improving surgical efficiency, and reducing the need for screw exchange.

Recently, a new electronic combined power tool and electronic depth gauge has been released for the market, i.e. Stryker CD NXT [7]. The measured bone depth is instantly shown on a large display when the drill encounters a loss of resistance, i.e., when the first and second bone cortex are breached. Moreover, it simultaneously shows the maximal drilling depth, which might be beneficial in surgical skill training to minimize the penetration depth of the drill bit beyond the second cortex, and thus soft tissue damage [8].

The electronic depth gauge is versatile and can be used with or without a soft-tissue protector as the relative distance of the retractable cannula around the drill bit is measured. To the best of our knowledge, the benefits and limitations of electronic depth in orthopedic surgery have not yet been scientificly explored.

This study aimed to determine whether an electronic depth gauge is found usefull by orthopaedic surgeons and compared it with a conventional manual depth gauge in a preclinical setting.

## Methods

In this preclinical randomized controlled, cross-over trial we tested the usability and acceptability of an electronic depth gauge compared with a conventional, manual depth gauge to determine screw length for orthopaedic internal fixations.

The study was conducted in the Department of Orthopaedics, Aarhus University Hospital, level 1 trauma center. A total of 45 participants with various levels of clinical experience were included, consisting of 14 orthopedic senior consultants, 8 orthopedic consultants, 11 orthopedic residents from the Department of Orthopaedic at Aarhus University Hospital, and 12 medical students from Aarhus University.

**Fig. 1:**
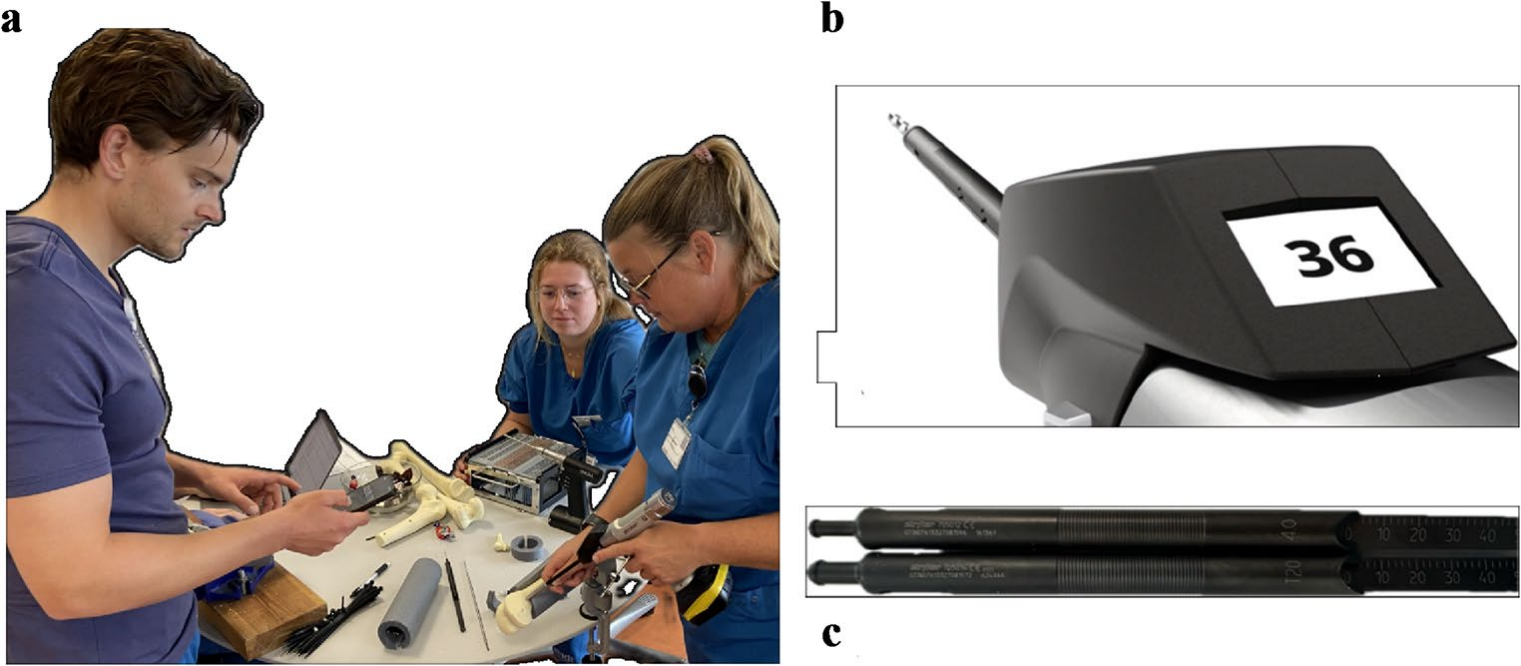
**a** illustrates the experimental setup. The participant is seen in the middle, and the instructor is seen to the left and is timing the procedure. The co-instructor is seen to the right and hands the screws. **b** illustrates the measured depth on the display of the Stryker CD NXT. **1c** illustrates the manual depth gauge used

The experimental setup is depicted in Fig. 1a-c.

**Fig. 2:**
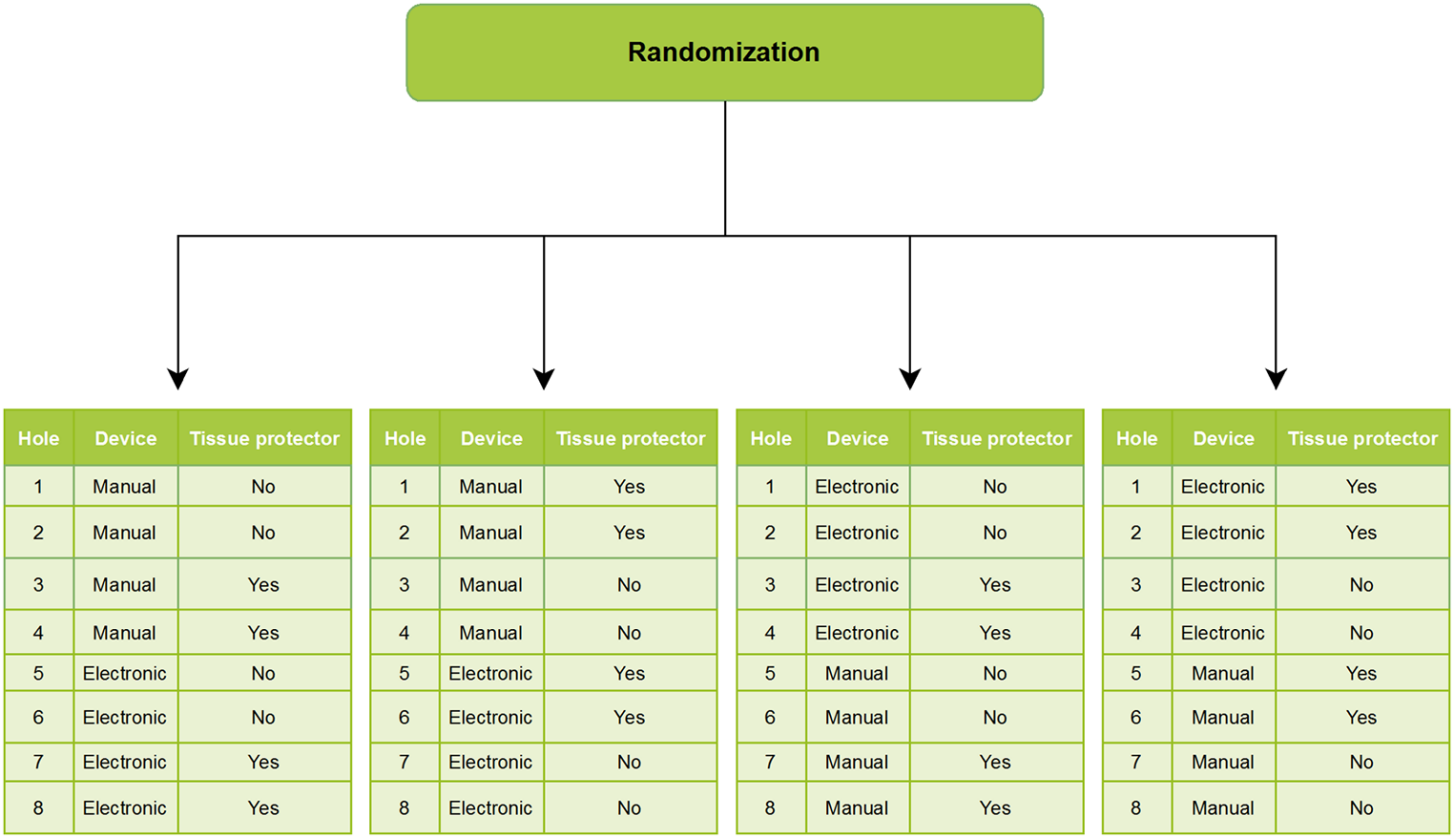
Randomization of participants. Participants were randomized to one of the four stings illustrated. Participants measured depth with the gauges in the following order: first four measurements with manual, then four measurements with electronic, or four measurements with electronic first, then four measurements with manual and soft tissue protector in the order no/no, yes/yes, no/no, yes/yes or yes/yes, no/no, yes/yes and no/no

Each participant was handed a written information sheet (Supplementary material Fig. 1 and Fig. 2) and oral instructions were given. All participants consented.

Each participant was asked to drill 8 holes, make 8 depth measurements, and insert 8 screws.

Each participant was randomized to the sequence “electronic -> manual” or “manual -> electronic” depth gauge and subsequently to the sequence “with -> without” or “without -> with” soft tissue protector. Please refer to Fig. 2 for an illustration of the randomization sequence.

Three different bones from *Sawbones USA* were used: proximal femur, femur, and tibia (SKU: 1130-21-33, 1100, and 1101). Sawbones were randomly selected between participants and participants were instructed to drill at random different locations of the sawbones to secure a varying bone thickness. The sawbones were mounted on a clamp. The far cortex, including the circumference of the sawbones, was covered in foam, so the penetration of the second cortex could not be seen nor digitally palpated (Fig. 1a).

A Ø3.2 mm drill bit was used (Stryker: 4607-099-032, 99 mm length without soft tissue protection or 4607-129-032, 129 mm length with soft tissue protector).

### Procedure with electronic depth gauge

Participants used the Stryker CD NXT according to the manufacturer’s instructions. After each drilling, the measurement shown on the display was noted, and participants were instructed to choose a screw length (Stryker, AXSoS3 TI 4.0 mm, cortical), screws were available in 2 mm increments. Afterwards, the screw was inserted. This procedure was then repeated for the next three screws resulting in 4 screws in total. The soft tissue protector and its longer drill bit were used for 2 of the holes according to the randomization. After 4 holes and 4 screws, the foam was removed and the far cortex was shown to the participants, and they were asked if they wanted to change the screw length, and if so to which size.

The penetration on the far cortex was measured using a ruler by an instructor and hidden from the participant. If screws did not penetrate the cortex and thus were not able to be measured with a ruler, penetration depth was noted as “negative”.

### Procedure with manual depth gauge

Participants also used the Stryker CD NXT, but the display of the electronic depth gauge was blinded using tape. After each drilling, the power tool was handed to an instructor, who noted the electronic measurement on the display, while participants remained blinded, and continued to measure the depth with a manual depth gauge (Stryker: 705012 4.0).

### Efficiency of the electronic vs. manual depth gauge

As a measure of efficiency, the time saving of the electronic depth gauge was assessed.

Using the manual depth gauge, we measured the time from far cortex penetration to the verbal request of screw length. When using the electronic depth gauge, there was no delay from electronic display of the measured depth to the verbal command regarding the measured screw length. The participants were unaware of the time consumption that was the subject of the assessment.

### System Usability Scale

**Fig. 3:**
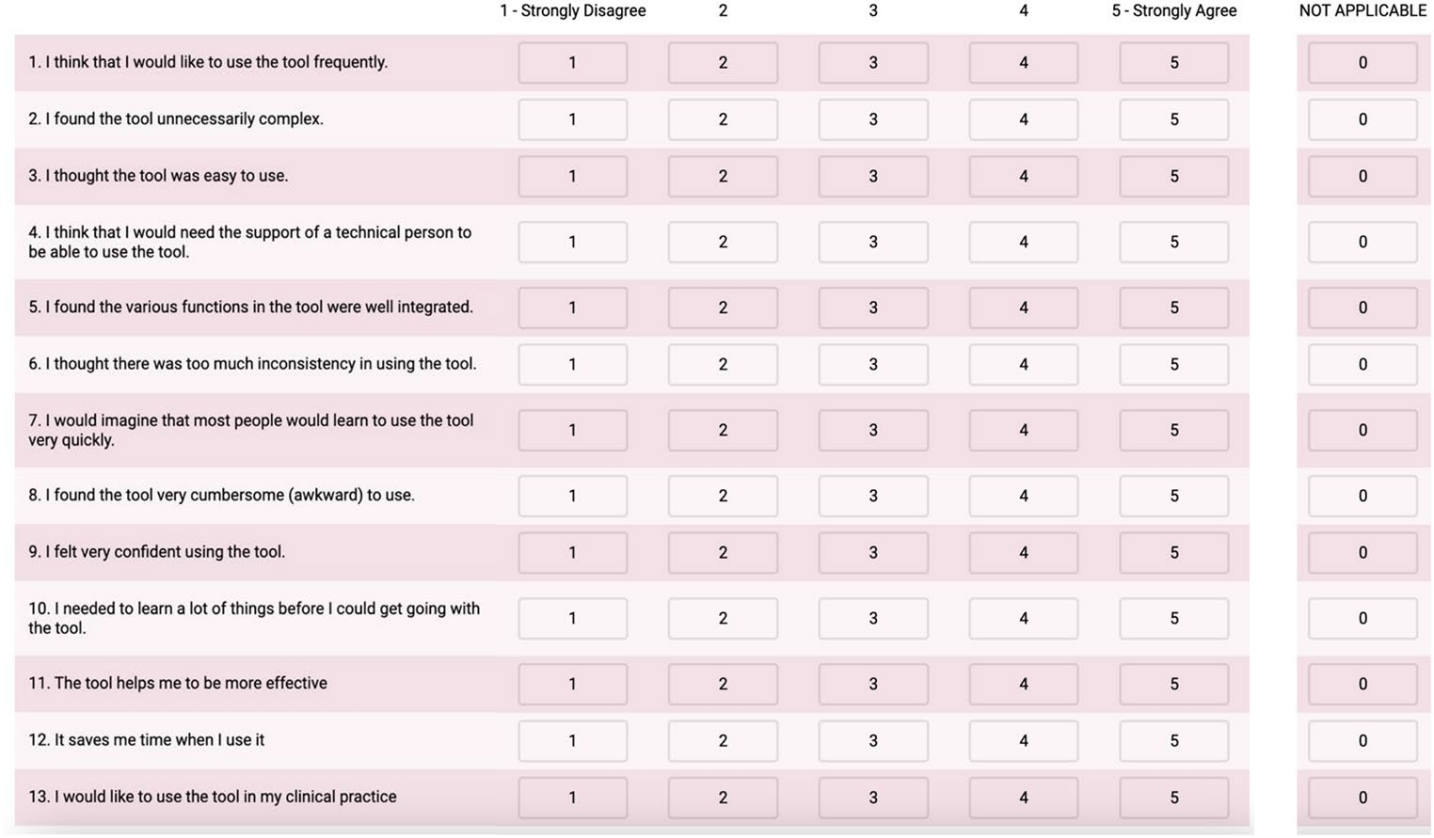
System Usability Scale (SUS)

Usability and acceptablility of the electronic depth gauge was assessed with the validated System Usability Scale (SUS) containing 10 items [9, 10] (Fig. 3). Each item is rated on a 5-point Likert scale with anchors: 1-strongly disagree and 5-strongly agree. The SUS score is calculated based on the following equation: 2.5*((Q1 + Q3 + Q5 + Q7 + Q9-5)+(25-Q2-Q4-Q6-Q8-Q10)). The full questionnaire is available as supplementary material. This methodology has been previously applied to measure the usability and acceptability of surgical devices [11–13].

For each of the following statements, please mark on box that best describes your reaction to the tool.

Willingness To Pay was estimated by asking participants for a relative amount, i.e. “How much do you think the hospital should be willing to pay to use the tool?”: Response options were: “The hospital should NOT be willing to pay”; Price of: 1, 2, 3, 4, 5, > 5 screws.

After finalizing data acquisition, a product specialist from Stryker informed the authors that the instructions for use recommend adding 2 mm to the measured depth when using the applied screws in combination with the electronic depth gauge. The participants were thus unaware of this during the trial and when completing the questionnaires. The results from the questionnaires are shown without incorporation of the addition of 2 mm. However, the conventional, manual depth gauge was intended for use for the applied Stryker AxSOS 3 titanium cortical screws Ø4.0 mm (reference number 705014). As shown in Fig. 1C, the depth gauge offsets the measured depth + 2 mm. Notably, this offset varies within various products of the same manufacturer and other vendors. As an example another manual depth gauge with 4 mm offset is also depicted in Fig. 1C.

Due to this new information, a post-hoc transformation of the screw penetration data was made to correct for the recommended adding of 2 mm when using the electronic depth gauge; 2 mm was added to all screw lengths measured with the electronic depth gauge. We decided to accept 0–3 mm protrusion. If screws inserted using the electronic depth gauge protruded less than 0 mm or more than 3 mm after the addition of the 2 mm, then it was deemed as “screw change likely”. If participants wanted to change the screw + 2 mm longer, then data was corrected to “no change”.

### Statistics

We did not perform a sample size calculation, because we did not have relevant information regarding the expected effect size. We therefore included as many consultants, junior doctors, and medical students as possible to test the electronic depth gauge.

Data on *time-saving* followed a non-gaussian distribution, after log-transformation data remained non-gaussian distributed. Therefore, the difference in time-saving between the three groups (student, resident, and consultant) was analyzed pair-wise using a Mann-Whitney test.

The remaining data followed a normal distribution, and an ANOVA with a Turkey post-test was used to assess the data.

### Ethical considerations and approval

Ethical approval was exempted according to Danish legislation and written approval by the regional ethical committee, reference number: REF 161/2024 (1-10-72-103-24). The study was conducted at the Department of Orthopaedics, a level I trauma center at Aarhus University Hospital. The study complied with the declaration of Helsinki and all participants granted their approval to use their pseudonymized data for publication. Written approval was also given by the individuals depicted in Fig. 1.

## Results

Complete data of all 45 participants were available for analysis. The demographic information of the participants is given in Table 1.

**Table 1:**
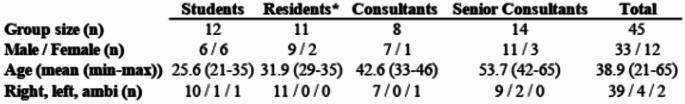
Demographics of participants

### Screw exchange

After post-hoc transformation of the screw lengths, there was no statistically significant association of use of manual vs. electronic depth gauge or the use of soft tissue protector (yes/no) with the rate of screw changes, *p* = 0.76 and *p* = 0.25 respectively (three-way ANOVA). In total 65/331 (20%) of screws were outside the predefined acceptable screw penetration range (0–3 mm) and thus at risk of being exchanged in the clinical setting.

Medical students changed 23/88 (26%) of screws, resident doctors 14/66 (13%), and consultants 33/176 (19%). The level of experience (student, resident, and consultant) was significantly associated with changing screws, *p* = 0.03 (three-way ANOVA). A Tukey post-test showed that residents changed significantly fewer screws than medical students, Tukey 95% CI 0.01–0.33. No significant differences between the remaining groups were found.

NB: The raw numbers of screw exchange before post-hoc transformation of data as described in the methods were: 160/331 (48%) exchanged screws. 31/160 (19%) of the screw changes were made when the manual depth gauge was used, and 129/160 (81%) when the electrical depth gauge was used.

### Efficiency of the electronic vs. manual depth gauge

A median time from penetration of the far cortex to the verbal request of screws i.e. *time-saving* of 21.3 s (IQR 14.1;32.3) for medical students, 12.2 s (IQR 8.7;23.3) for residents, and 13.5 s (IQR 10.6;17.3) for consultants were found. Thus, in terms of efficiency, participants across all levels of experience saved more than 10 s per screw using the electronic depth gauge. Medical students saved significantly more time than residents doctors and consultants, *p* < 0.001 and *p* < 0.001 respectively.

### Usability

The median SUS score of all participants (*N* = 45) was 87.5 (min. 57.5; IQR 80-92.5; max. 100).

**Fig. 4:**
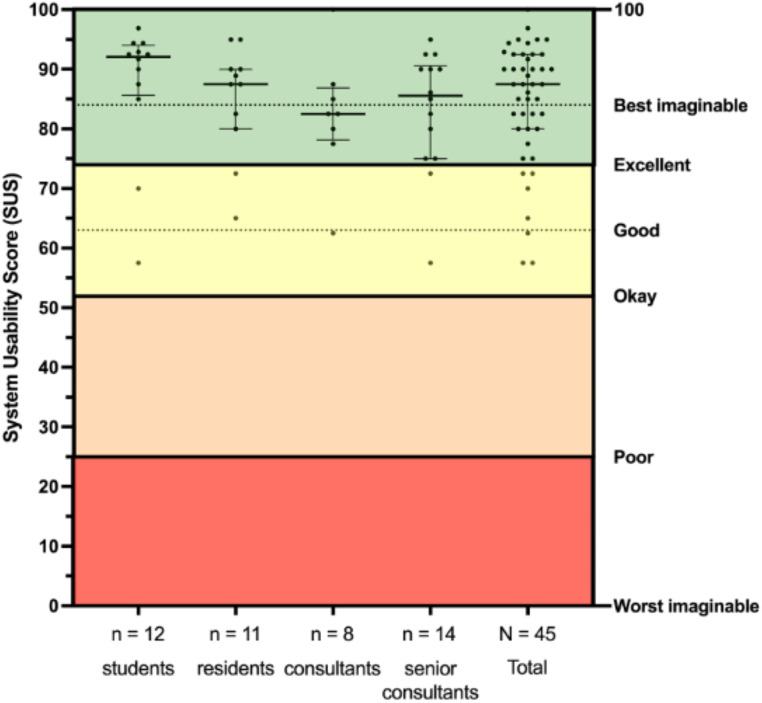
System Usability Score (SUS). Individual SUS scores, median, and interquartile range (error bars) are depicted subdivided by the level of experience. Adjectives interpreting SUS scores are stated on the right Y-axis including colored backgrounds and dotted lines for “best imaginable” and “good” thresholds

One-way ANOVA did not find any statistically significant differences between the four groups of participants (*p* = 0.84), consequently, the total score of all participants is stated below. However, for details regarding the SUS score of the four different groups of participants, please refer to Fig. 4.

Of the 45 participants, 28 + 10 = 38 (84%) participants rated the tool’s usability as “best imaginable” / “excellent”, while 4 participants rated the tool “Good” and only 3 participants as “Okay”. No participants gave the tool the adjective “poor” or “worst imaginable”. Correspondingly, 40/45 participants’ SUS scores deemed the tool “acceptable”, and the remaining 5/45 scores were “marginally acceptable”.

The willingness to pay among the participants ranged from “the hospital should not pay extra for the device” to more than the equivalent of 5 screws. The median number of screws was 2.5 (0; 1–4;>5). Notably, 5 respondents stated that the hospital should not be willing to pay.

In total, 34 participants stated free text comments regarding “the most positive” and 28 coments regarding “the most negative” aspects of the tool. The data was analyzed and summarized in Table 2. Highlighted responses include the safety functions, e.g. “Display shows?? if not responding properly, e.g. if the drill guide is lifted from the bone surface, etc.” and the need for manual adjustment to adjust for the purpose of the screw, i.e. use with / without a plate and manual adjustment for plate thickness.

**Table 2:** Thematic summary of participants’ free text comments regarding the tool

Most positive aspects (*N* = 34)	Most negative aspects (*N* = 28)
Time saving (*n* = 18)	Consistently 2 mm too short (*n* = 17)
Easy to use (*n* = 18)	Lack of trust in the tool: real depth? (*n* = 4)
Accurate (*n* = 6)	Manual adjustment regarding screw length is needed depending on with / without a plate, plate thickness (*n* = 4)
Safety (*n* = 4)	Expected price (*n* = 4)

## Discussion

This study aimed to evaluate the usability and acceptability of an automated electronic depth gauge compared with a conventional manual depth gauge for determining screw length in orthopedic surgery. These findings indicate that the electronic depth gauge offers significant advantages in terms of efficiency and usability.

The electronic depth gauge significantly reduced the time required to measure screw length across all levels of clinical experience. Medical students, residents, and consultants all saved more than 10 s per screw using the electronic device, with medical students showing the greatest time savings. The significant efficiency gain for untrained students is consistent with previous studies that have highlighted a positive association between the level of surgical training and efficiency in depth gauge measurement [2]. However, the saved time per screw is not likely to be clinically relevant. Time-saving may become clinically relevant if placing many screws, e.g. multi-traumatized patients needing extensive internal fixation. Exchanging screws on the other hand may compromise screw fixation and should thus be minimized. Interestingly, the rate of screw changes was higher when using the electronic depth gauge compared to the manual gauge before post-hoc data transformation. This discrepancy is attributed to the initial lack of awareness regarding the need to add 2 mm to the measured depth when using the electronic gauge, as recommended by the manufacturer’s instructions. After adjusting for this factor, the rate of screw changes decreased, suggesting that proper training and awareness are crucial for optimizing the use of electronic depth gauges. However, approximately 20% of all screws still fell out of the accepted range of penetration (0–3 mm). Use of the electronic depth gauge thus does not necessarily reduce the number of screw exchanges in a clinical setting.

Despite these findings, the vast majority of the 45 participants were satisfied with the power tool with incorporated depth gauge and rated its usability as excellent, i.e. median System Usability Scale (SUS) score of 87.5. Even thoug changing 129/168 (77%) of screws when the electronic depth gauge was used, the majority of participants rated the tool as “best imaginable” or “excellent,” and no participants rated it as “poor” or “worst imaginable”. These findings suggest that the electronic depth gauge has great user satisfaction and acceptance.This is unlike other technological aids, for instance the ADAPT system for intertrochanteric nailing of femoral fractures, which some of the participants of this study have previously rated as “unacceptable” [11]. This also partly refuses the argument that surgeons have a positive attitude towards new tools, which may positively bias the reported usability.

Overall, the literature on the use of electronic depth gauges in orthopedic surgery is sparse since the technology is new and rarely applied in the clinical setting. Here, we report no difference in the rate of screw exchange when an electronic depth gauge is compared to a manual depth gauge.

The literature on how clinical experience affects the accuracy of drilling and screw placement in orthopedic surgery is conflicting [1, 2, 5, 14, 15]. Our findings contribute to this discussion by showing that resident doctors changed significantly fewer screws than medical students, yet their performance was comparable to that of consultants. This observation may be attributed to the Danish medical training system, where orthopedic resident doctors have a high volume of fracture cases and surgical procedures early in their careers [16], which may rapidly improve their technical skills.

### Strengths and limitations

One major limitation is the difference in measuring the actual depth and the screw length needed, which depends on the purpose of the screw, such as compression or use with a plate of varying thickness. Many participants reported that the electronic tool consistently measured a shorter screw length, which can be attributed to the manual depth gauge’s offset of + 2 mm. This discrepancy highlights the importance of understanding these differences to save time and cost. The 2 mm has been incorporated into the manufacturer’s instructions for the electronic depth gauge. Additionally, the use of sawbones without a soft-tissue envelope may affect the tool’s reliability, as it does not fully replicate clinical conditions, and engaging of hook of the manual depth gauge in the far cortex of the bone may be easier. A thick periosteum, osteoporosis, or a comminuted fracture also add to the difficulty to engage the hook of a manual depth gauge in clinical practice. The time saving is thus likely to be underestimated in the present study.

Importantly, the versatility of the electronic depth gauge should be increased in order to be helpful in the clinical setting. The current version cannot be used with longer drill sleeves as commonly used when locking intramedullary nails. Moreover, the electronic depth gauge is sold as “single use only”, while manual depth gauges can be used sterilized. Consequently, the sustainability of the product could be improved if re-sterilization and use in multiple patients were made feasible.

This study has several strengths: First, the randomized controlled, cross-over study design enhances the reliability of the findings. Inclusion of 45 participants with various levels of clinical experience provides a comprehensive evaluation. Clear and uniform written participant information and verbal prompts ensured internal consistency. Data collection by the same individuals within a three-day period minimized variation in instructions. Blinding of the far cortex by a simulated soft tissue envelope impaired visual and tactile assessment of penetration, enhancing the study’s rigor.

## Conclusion

Orthopaedic consultants, residents and medical students deemed the use of the electronic depth gauge “excellent” reflected by a high SUS score. The electronic depth gauge was time-efficient when measuring the depths, but it did not significantly reduce screw exchanges compared to manual depth measurement in the present study.

Proper training and awareness of device-specific recommendations are essential in order to choose the correct offset of the measured and displayed bone depth compared to the screw system, e.g. +0, 2, 4 mm depending on the medical implant.

*During the preparation of this work*,* the authors used the AI-tool Microsoft Copilot in order to improve sentencing. After using this tool/service*,* the authors reviewed and edited the content as needed and takes full responsibility for the content of the publication.*

## Electronic supplementary material

Below is the link to the electronic supplementary material.

Supplementary Material 1

## Data Availability

No datasets were generated or analysed during the current study.
