# Supplementary material for: Usability and acceptability of a power tool with electronic depth gauge for orthopedic drilling – a preclinical randomized controlled trial in sawbones
Source: Arch Orthop Trauma Surg. 2025 Apr 11;145(1):237. doi: 10.1007/s00402-025-05839-3 (PMC11991983; doi:10.1007/s00402-025-05839-3)
Supplement: Supplementary file 1 — Supplementary Material 1 [file 402_2025_5839_MOESM1_ESM.docx]

**Supplementary**

Figure 1 illustrates the actual written information handed to the participants before participation in Danish. Figure 2 illustrates the written information translated into English.

**Figure 1**

**Figure 2**
